# Characterization of protein lactylation in healthy and ischemic mouse hearts

**DOI:** 10.3389/fcvm.2025.1644886

**Published:** 2025-09-29

**Authors:** Daiqian Wu, Yuanjuan Tang, Xingbing Li, Shiqiang Xiong, Zhen Zhang, Jinjuan Fu

**Affiliations:** ^1^ Department of Cardiology, Chengdu Cardiovascular Disease Research Institute, Affiliated Hospital of Southwest Jiaotong University, The Third People's Hospital of Chengdu, Chengdu, China; ^2^ Division of Cardiology, The First Affiliated Hospital of Chongqing Medical University, Chongqing, China

**Keywords:** myocardial infarction, lactylation, lactate, posttranslational modification, proteomics, lactylome

## Abstract

**Background:**

Recent findings highlight the growing importance of protein lactylation, a modification driven by lactate, in healthy and diseased states. However, its significance in myocardial infarction (MI) remains unclear. Here, we characterized lactylation in healthy and ischemic hearts, revealing its profound implications.

**Methods:**

Global proteomics and lactylome profiling were conducted on the hearts of healthy mice and mice with induced MI. Protein expression analysis, enrichment analysis, cellular compartment analysis, and protein-protein interaction network construction were conducted to identify potential molecular features. The changes in total protein lactylation levels and the lactylation levels of identified representative proteins in healthy and ischemic hearts were validated.

**Results:**

Proteomic analysis revealed that MI led to a metabolic shift from oxidative phosphorylation and fatty acid *β*-oxidation toward hypoxia-induced glycolysis. Western blotting and immunofluorescence analyses conclusively demonstrated the presence of protein lactylation in healthy hearts, with significantly elevated lactylation levels following MI. Lactylome profiling identified 1,674 lactylation sites across 477 cardiac proteins under physiological conditions, with 44.03% (210/477) being singly-lactylated proteins. Myosin-6 and titin were identified as the proteins having the most lactylation sites in the heart. Comparative analysis revealed 61 upregulated lactylation sites across 53 proteins and 30 downregulated sites across 27 proteins in infarcted hearts relative to healthy controls. Functional enrichment analyses suggested that proteins with altered lactylation modification post-MI mainly included metabolic enzymes, cytoskeletal proteins, and RNA binding proteins. We created lactylation modification maps for these three types of proteins in ischemic hearts.

**Conclusions:**

We present the first comprehensive lactylation atlas in healthy and ischemic mouse hearts, offering new avenues to explore MI and cardiovascular diseases.

## 1 Introduction

Myocardial infarction (MI), a common condition caused by prolonged ischemia of coronary arteries, remains a leading cause of death worldwide. Coronary occlusion significantly reduces the oxygen supply to cardiac cells, resulting in decreased oxidative phosphorylation and an enhanced glycolytic rate (1, 2). This metabolic shift leads to increased lactate production, aligning with both clinical and animal studies that have reported elevated circulating lactate levels after MI (3, 4). Lactate is far more than a mere byproduct of glycolysis. Beyond serving as a vital energy source for cardiomyocytes, it also plays crucial roles in maintaining homeostasis and supporting various physiological functions (5). What is even more remarkable is that lactate can act as a substrate for epigenetic modifications, thereby influencing gene transcription and protein function (6). Protein lysine L-lactylation (Kla), or called lactylation, is a recently discovered type of protein posttranslational modification (PTM) (7). Induced by L-lactate, lactylation is associated with hypoxia and the Warburg effect, participating in a range of physiological and pathological processes, including development, cancer, and cardiovascular diseases (6, 8). It has been reported that lactylation of Snail1 can promote cardiac endothelial-to-mesenchymal transition (EndoMT) and worsen cardiac fibrosis after MI (4). On the contrary, another study revealed that lactate and lactylation play a beneficial role in cardiac repair after MI, as Histone H3 K18 lactylation (H3K18la) in monocytes promotes the expression of cardiac repair genes, such as Lrg1, Vegf-a and IL-10 (9). Therefore, the relationship between lactylation and MI has not yet been fully elucidated. Understanding the status of protein lactylation before and after cardiac injury may hold great potential for the treatment of MI.

To explore the biological importance of Kla in healthy and injured heart, global lactylome and proteome analyses were performed using a robust label-free quantification approach. This comprehensive characterization of Kla could open up new avenues for research on MI and other cardiovascular diseases.

## 2 Materials and methods

### 2.1 Animals

All animal experiments were carried out according to the Guide for the Care and Use of Laboratory Animals of the National Institutes of Health, and the ARRIVE guidelines 2.0. C57BL/6J mice were purchased from the Experimental Animal Center of the Daping Hospital. The experimental protocol was approved by the Third Military Medical University Animal Use and Care Committee (AMUWEC20247120).

### 2.2 Construction of MI model

MI was induced in 8-week-old male mice through permanent ligation of the proximal left anterior descending (LAD) coronary artery, as previously described (10). Briefly, mice were maintained under anesthesia with 2% isoflurane inhalation delivered in 100% oxygen flow, followed by an incision made between the third and fourth intercostal spaces. After exposing the heart, the LAD was ligated with a 7-0 silk suture, positioned 2–3 mm distal to the left atrial appendage. The thoracic cavity was closed with 6-0 silk sutures, and the mice were then extubated. Sham-operated mice, which underwent the same procedure as the MI group except for the ligation of the LAD, served as the control group. After 3 days of MI induction, the hearts were collected for analysis.

### 2.3 Protein extraction and trypsin digestion

The heart tissue samples were taken from −80 °C and placed into a mortar pre-chilled with liquid nitrogen. Liquid nitrogen was added, and the samples were finely ground into a powder. Each group of samples was then mixed with four times the volume of lysis buffer (50 µM PR-619 (Cat. #HY-13814, MedChemExpress), 1% Triton X-100 (Sangon Biotech), 50 mM Nicotinamide (Cat. #HY-B0150, MedChemExpress), 10 mM dithiothreitol (Sigma-Aldrich), 1% protease inhibitor cocktail (Merck Millipore), 3 µM Trichostatin A (Cat. #HY-15144, MedChemExpress) and 2 mM EDTA(Sigma-Aldrich)) (11), followed by sonication for cell lysis. After centrifugation at 12,000 g for 10 min at 4 °C, the supernatant was transferred to a new centrifuge tube, and protein concentration was measured by an enhanced BCA protein assay kit (Cat. #P0010, Beyotime). Equal amounts of protein from each group were taken, and Trichloroacetic acid (Sigma-Aldrich) was slowly added to reach the final concentration of 20%, followed by vortex mixing. After 2 h of precipitation at 4 °C, the samples were centrifuged at 4,500 g for 5 min. The pellet was washed three times with acetone and air-dried. Then, 200 mM Tetraethylammonium bromide (Sigma-Aldrich) was added, and the mixture was sonicated. Trypsin [0.1 μg/μl; trypsin: protein = 1:50 (m/m)] was added for overnight digestion. Dithiothreitol (Sigma-Aldrich) was then added to a final concentration of 5 mM, and the mixture was reduced at 56 °C for 30 min. Subsequently, iodoacetamide (Sigma-Aldrich) was added to a final concentration of 11 mM, and the samples were incubated in the dark at room temperature for 15 min.

### 2.4 Lactylated peptide enrichment

The peptides were dissolved in immunoprecipitation (IP) buffer (100 mM NaCl, 1 mM EDTA, 50 mM Tris-HCl, 0.5% NP-40, pH = 8.0) and the supernatant was transferred to pre-washed resin (Cat. #PTM1404, PTM Bio) (12). The mixture was gently shaken for overnight incubation on a rotating shaker at 4 °C. After incubation, the resin was washed four times with IP buffer and twice with deionized water. Finally, the peptides bound to the resin were eluted with 0.1% trifluoroacetic acid elution buffer. For liquid chromatography-tandem mass spectrometry (LC-MS/MS) analysis, the resulting peptides were desalted with C18 ZipTips (Millipore) according to the manufacturer's instructions.

### 2.5 LC-MS/MS analysis

The peptides were dissolved in mobile phase A of the liquid chromatography and then separated using the NanoElute ultra-high-performance liquid chromatography (UHPLC) system (Bruker Daltonics). The mobile phase consisted of solvent A (0.1% formic acid, 2% acetonitrile in water) and solvent B (0.1% formic acid in acetonitrile). The liquid chromatography gradient settings were as follows: 0–18 min, 6%–22% B; 18–22 min, 22%–30% B; 22–26 min, 30%–80% B; 26–30 min, 80% B, with a flow rate maintained at 500 nl/min. After separation by the UHPLC system, the peptides were subjected into the Capillary Electrospray Source for ionization and then analyzed by the timsTOF Pro mass spectrometer for data acquisition (13). The electrospray source voltage was set to 1.6 kV, and both the peptide precursors and their secondary fragments were detected and analyzed using time-of-flight (TOF). The data acquisition mode used was data-independent parallel accumulation serial fragmentation (dia-PASEF). The range for the primary mass spectrum scan was set from 100–1,700 m/z. After acquiring one primary mass spectrum, 8PASEF mode acquisitions were performed. The secondary mass spectrum scan range was set from 425–1,025 m/z, with a window of 25 m/z for each scan.

### 2.6 Database search

Raw mass spectrometry data were searched against a Swissprot protein sequence database (Mus_musculus_10090_SP_20230103.fasta) by Spectronaut (v17). A maximum of four missing cleavages was allowed for trypsin/*P* digestion. Cysteine alkylation was considered a fixed modification. Variable modifications were methionine oxidation, N-terminal acetylation and lysine lactylation. For further data filtering, the identification result filtering criteria were set as follows: Precursor and protein false discovery rate (FDR) were set to 1%; identified proteins must contain at least one unique peptide.

### 2.7 Bioinformatics analyses of proteomic

GO enrichment analysis and KEGG pathway analysis were performed using the Database for Annotation, Visualization, and Integrated Discovery (DAVID). GSEA was performed with GSEA v4.3.3 software. The motif characteristics of the modification sites were analyzed by MoMo analysis tool (http://meme-suite.org/tools/momo) based on the motif-x algorithm. The protein interaction relationships were obtained by comparing with the STRING protein interaction network database (https://string-db.org/; v12.0), and then visualized using the Cytoscape v3.7.2 tool. The subcellular localization of proteins was annotated using the WolF PSORT software (http://www.genscript.com/psort/wolf_psort.html).

### 2.8 Lactate measurement

Heart tissue lactate levels were quantified using a Lactate Assay Kit (Cat. #A019-2, Jiancheng Bio, Nanjing, China) following the manufacturer's instructions. Briefly, tissues were homogenized in four volumes of lactate assay buffer and centrifuged to obtain the soluble supernatant for analysis. The assay procedure was performed as follows: the sample, enzyme working solution, and chromogenic agent were sequentially added to the reaction mixture. After vortex mixing, the reaction was incubated at 37 °C for 10 min and then terminated by adding stop solution. Absorbance was measured at 530 nm using a cuvette with a 1 cm light path.

### 2.9 Immunofluorescence staining

Paraffin sections were deparaffinized, and then were put in sodium citrate antigen repair buffer (Cat. #P0083, Beyotime) for high-temperature antigen repair for 30 min. The sections were blocked with blocking solution (Cat. #P0260, Beyotime) for 60 min at room temperature and then treated with antibodies against pan-Kla (Cat. #PTM-1401RM, PTM Bio) and Cardiac Troponin T (cTNT, Cat. #MA5-12960, Invitrogen) overnight at 4 °C. The sections were then washed with PBS thrice and incubated with Alexa Fluor 488 and 555 secondary antibodies (Invitrogen) for 60 min in the dark at 37 °C.

### 2.10 Western blotting (WB) and immunoprecipitation (IP)

Mouse heart tissues were homogenized in lysis buffer [Protease inhibitor cocktail III (Cat. #535140, Merck Millipore, 1:200), Protease inhibitor Cocktail IV (Cat. #524633, Merck Millipore, 1:200), Protease inhibitor Cocktail V (Cat. #539137, Merck Millipore, 1:60), 50 μM PR-619 (Cat. #HY-13814, MedChemExpress), 50 mM nicotinamide (Cat. #HY-B0150, MedChemExpress) and 3 μM Trichostatin A (Cat. #HY-15144, MedChemExpress)]. For WB, the proteins samples were separated by sodium dodecyl sulfate polyacrylamide gel electrophoresis (SDS-PAGE) and transferred to a Nitrocellulose Membrane (Cat. #66485, Pall Corporation). After 1 h of incubation in 5% nonfat milk (Cat. #P0216, Beyotime), the membranes were incubated with specific primary antibodies at 4 °C overnight and appropriate secondary antibodies (Li-Cor, IRDye 800 CW, 1:10000) for 1 h at room temperature next day. For IP, the hearts were homogenized in above-mentioned prepared lysis buffer. The lysates were centrifuged at 12,000 g for 15 min at 4 °C. The supernatant was collected and the extracted protein was determined with BCA Protein Assay kit (Beyotime). The pre-washed 50ug Protein A/G magnetic beads (Cat. #HY-K0202, MedChemExpress) were mixed with 5 μg anti-Pan Kla antibody (Cat. #PTM-1401RM, PTM Bio, RRID: AB_2942013) at room temperature for 1 h. Then, 1 mg of protein lysate and the antibody-conjugated beads complex were incubated 4 °C overnight on an orbital shaker. The supernatant was collected next day, and the magnetic beads bound with protein were washed three times with PBST (PBS containing 0.1% Tween-20). Then the magnetic beads were separated, and 50 μl 1 × SDS-PAGE Loading Buffer was added to the magnetic beads and heated at 95 °C for 5 min. For IP negative controls, only 1/40 of each sample was used. Finally, the protein was detected using immunoblotting. The following antibodies were used for WB and IP: rabbit anti-Pan Kla (Cat. #PTM-1401RM, PTM Bio, 1:1,000 for WB, 5 μg for IP), rabbit anti-CS (Cat. #ab96600, Abcam, RRID: AB_10678258, 1:1,000), mouse anti-PFKL (Cat. #68385-1-Ig, Proteintech, RRID: AB_3085103, 1:1,000), mouse anti-MYH6 (Cat. #sc-32732, Santa cruz, RRID: AB_670118, 1:200), rabbit anti-CSRP1 (Cat. #sc-390418, Santa cruz, 1:100), rabbit anti-PDLIM1 (Cat. #11674-1-AP, Proteintech, RRID: AB_2161631, 1:1,000), rabbit anti-DDX5 (Cat. #F1128, Selleck, 1:1,000), rabbit anti-SARNP (Cat. #15798-1-AP, Proteintech, RRID: AB_1959356, 1:1,000), mouse anti-GAPDH (Cat. #60004-1-Ig, Proteintech, RRID: AB_2107436, 1:10,000).

### 2.11 Statistical analysis

Statistical analyses were performed using GraphPad Prism (version 9.5.1) or R (version 4.3.3). The procedure for quantification of proteomic data was referenced from a previous study (14). Briefly, protein/peptide intensities were normalized across all samples to generate normalized intensity values. Subsequently, a centering transformation was applied to these normalized intensities to obtain relative quantification values. The relative quantification value of each modification site was divided by the relative quantification value of its corresponding protein to eliminate the effect of protein expression on the observed modification levels. The fold change (FC) between two comparison groups was then defined as the ratio of the mean quantification values for each protein or peptide. Prior to statistical analysis, all relative quantification values were log2-transformed to ensure the data approximated a normal distribution. Then the normality of data distribution was assessed using the Shapiro–Wilk test, and the homogeneity of variances was assessed using Levene's test. For comparisons that met both the assumptions of normality and equal variance, Student's *t*-test was applied; otherwise, the Welch's *t*-test was used. Additionally, for the proteomics data, FDR correction was applied to *p*-values via the Benjamini-Hochberg procedure, and the resulting q-values were calculated and presented. All quantification graphs in this study are presented as the mean ± S.D. A *p*-value < 0.05 was considered statistically significant.

## 3 Results

### 3.1 Proteomic analysis revealed metabolic reprogramming in post-MI hearts

**Figure 1:**
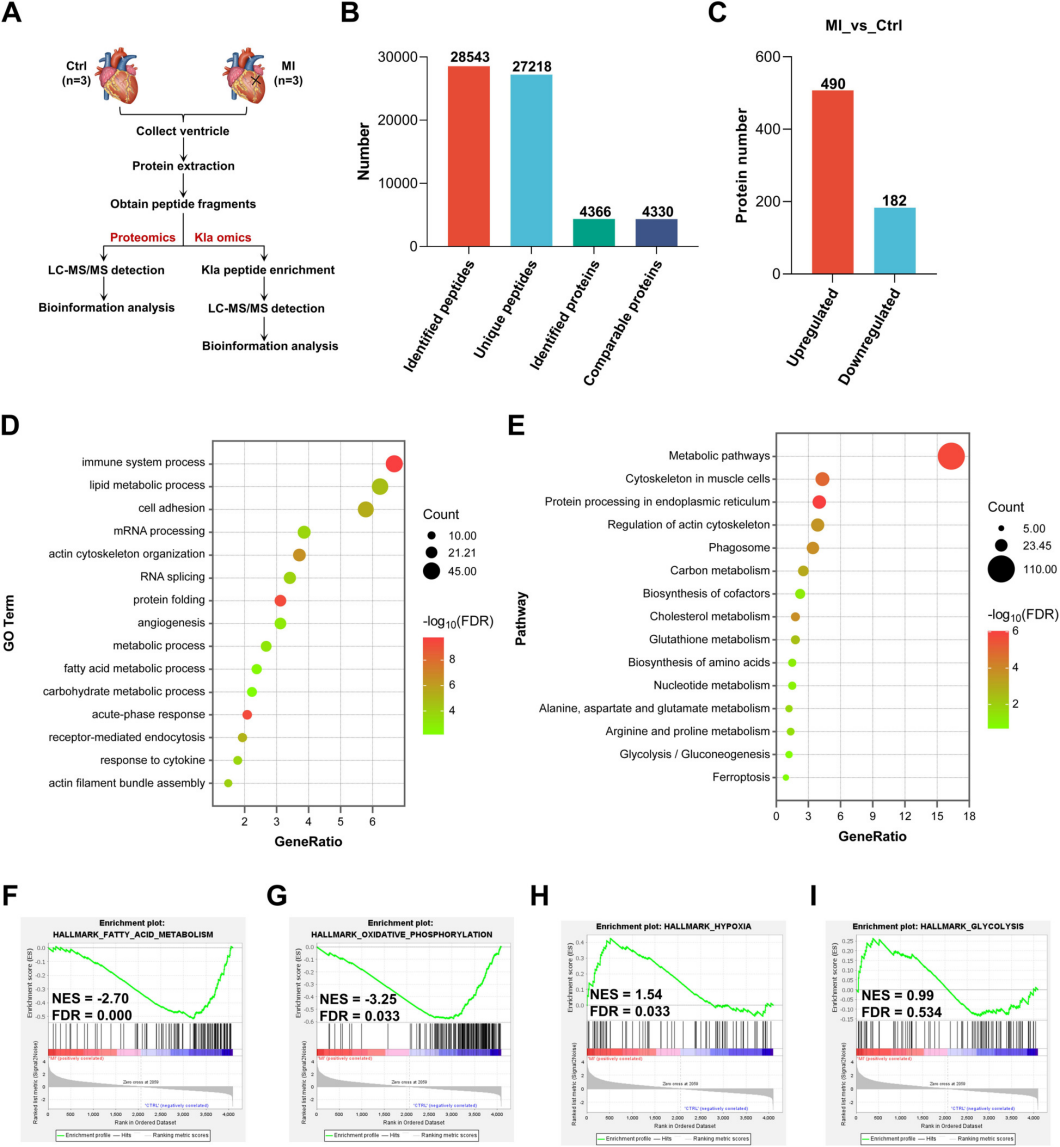
Proteomic analysis revealed metabolic reprogramming in post-MI hearts. **(A)** Schematic representation of proteomics and Kla omics. **(B)** Summary of identified peptides, unique peptides, identified proteins and comparable proteins. **(C)** Summary of differentially expressed proteins post-MI. **(D)** GO enrichment analysis of all the differentially expressed proteins. **(E)** KEGG pathway analysis of all the differentially expressed proteins. **(F–I)** GSEA showed the downregulation of genes involved in fatty acid metabolism and oxidative phosphorylation, together with upregulation of genes involved in hypoxia and glycolysis post-MI (relative to the control group). Kla, lysine L-lactylation; NES, normalized enrichment score; GO, gene ontology; KEGG, Kyoto encyclopedia of genes and genomes; GSEA, gene set enrichment analysis; FDR, false discovery rate.

To elucidate the pathophysiological processes underlying MI in mouse hearts, we performed comprehensive proteomic profiling to compare global protein expression patterns between sham-operated controls and infarcted hearts (Figure 1A). We totally identified 28,543 peptides and 4,366 proteins, in which 4,330 proteins were comparable (Figure 1B, Supplementary Material S1). By setting FC > 1.5 or FC < 1/1.5, with a *p*-value < 0.05, 490 proteins were upregulated and 182 proteins were downregulated 3 days post-MI (Figure 1C, Supplementary Material S2). We then performed Gene Ontology (GO) enrichment analysis and Kyoto Encyclopedia of Genes and Genomes (KEGG) pathway analysis of all the differentially expressed proteins. The results of GO enrichment analysis suggested that some biological processes were influenced in post-MI hearts, such as immune system process, kinds of metabolic processes, RNA processing, angiogenesis and acute-phase response (Figure 1D). KEGG pathway analysis showed that the most significant change was relation to metabolic pathways in post-MI hearts (Figure 1E). Subsequently, we asked the status of metabolic pathways through Gene Set Enrichment Analysis (GSEA), then found that fatty acid metabolism and oxidative phosphorylation were significantly inhibited (Figures 1F,G), whereas hypoxia-related pathway and glycolysis tended to be activated instead (Figures 1H,I). Collectively, these results defined metabolic remodeling as a central pathophysiological feature of post-MI hearts, featuring coordinated downregulation of fatty acid metabolism and oxidative phosphorylation, concomitant with induction of glycolysis.

### 3.2 The global Kla level was increased in post-MI hearts

**Figure 2:**
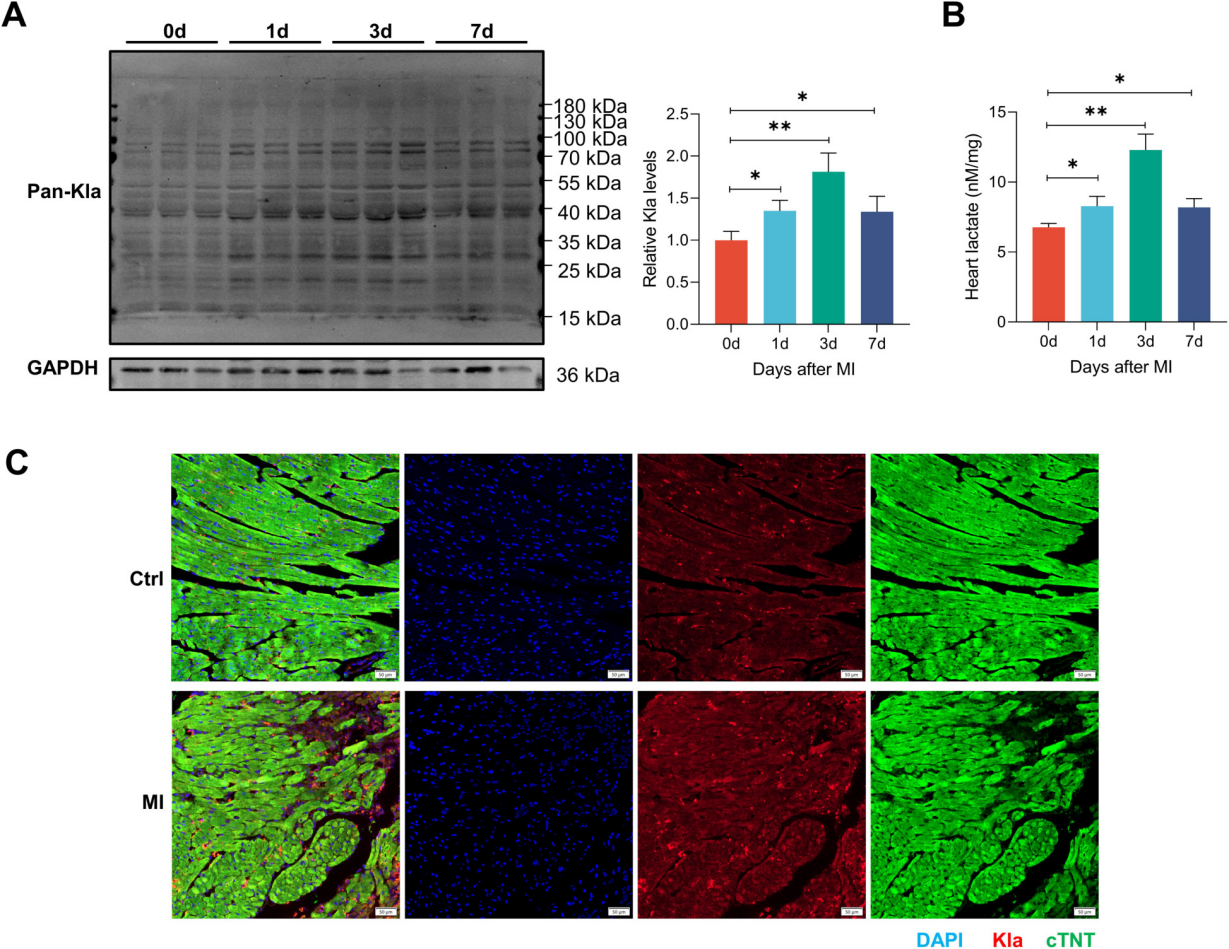
The global Kla level was increased in post-MI hearts. **(A)** The global lactylation levels in MI mice were detected by western blotting (*n* = 3). The right panel showed the results of the quantified analysis. **(B)** Cardiac lactate levels were measured by commercially available kit (*n* = 3). **(C)** Immunofluorescence staining of Pan-Kla (red) and cTNT (green) in control and MI (3 days post-MI) groups (scale bar = 50 μm). *P < 0.05, **P < 0.01.

Previous studies have revealed that MI can increase circulating lactate levels (3, 4). As glycolysis increases in infarcted heart, we directly measured the total protein lactylation status together with the lactate levels of heart tissues. We observed that the abundance of lysine lactylation and lactate both increased slightly in infarcted hearts one day post-MI, peaked at three days post-MI, and then declined (Figures 2A,B). Immunofluorescence staining of cardiac tissues also confirmed an overall increase in protein lactylation modifications three days post-MI (Figure 2C). Therefore, in the subsequent study, we identified Kla sites in both the control group and MI group, aiming to explore the pattern of this modification under physiological conditions and disease states.

### 3.3 Identification of Kla in the healthy mouse heart

**Figure 3:**
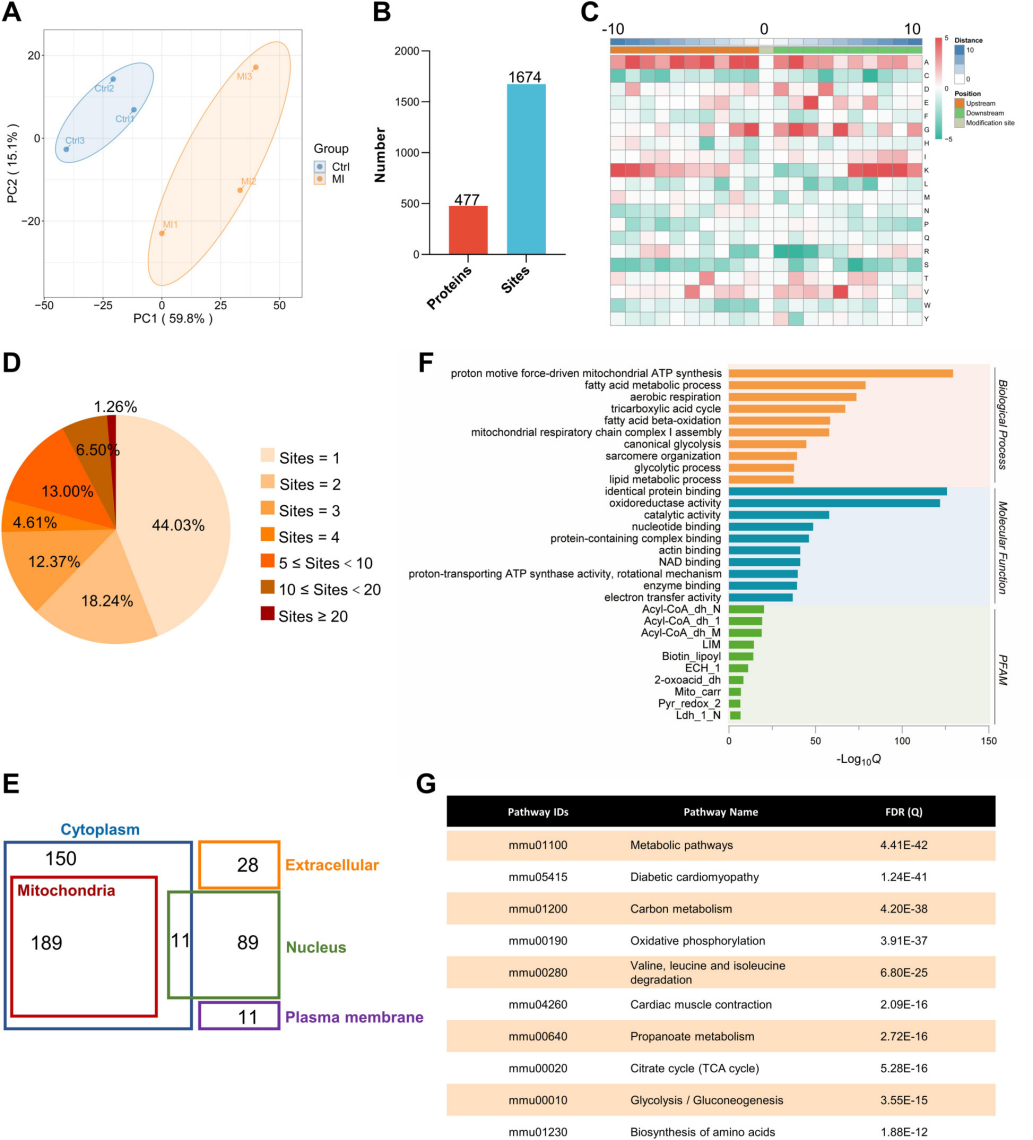
Identification and functional analysis of Kla sites in healthy heart. **(A)** PCA with a scatterplot of the first (PC1) and second (PC2) principal components was shown (*n* = 3 per group). **(B)** Summary of identified lactylated proteins and lactylation sites. **(C)** The frequency of amino acids surrounding lactylated lysines was analyzed, with 10 amino acids taken upstream (−10 on the *x*-axis) and 10 amino acids taken downstream (+10 on the *x*-axis), and the target lysine itself positioned at 0 on the *x*-axis. **(D)** Distribution of the number of lactylation sites per protein. **(E)** Cellular compartment distribution of 477 quantified lactylated proteins in our dataset predicted by Wolf PSORT. **(F)** The bar graphs showed representative ontology annotations enriched in the lactylated proteome. **(G)** KEGG pathway analysis of all the identified lactylated proteins. PCA, principal component analysis.

Figure 1A illustrates the workflow of our Kla omics analysis. Eight-week-old mice were subjected to control or MI treatment. Proteins from left ventricular tissues were harvested and Kla proteins were purified by IP targeting lactylated lysines and detected through LC-MS/MS. Based on proteomics and Kla omics analyses, Kla levels at each site were detected by normalizing Kla peptide abundance with protein abundance. The principal component analysis (PCA), which was performed to validate the quality of the MS data, showed that the two groups, control and MI groups, were separated into two distant quadrants (Figure 3A).

**Figure 4:**
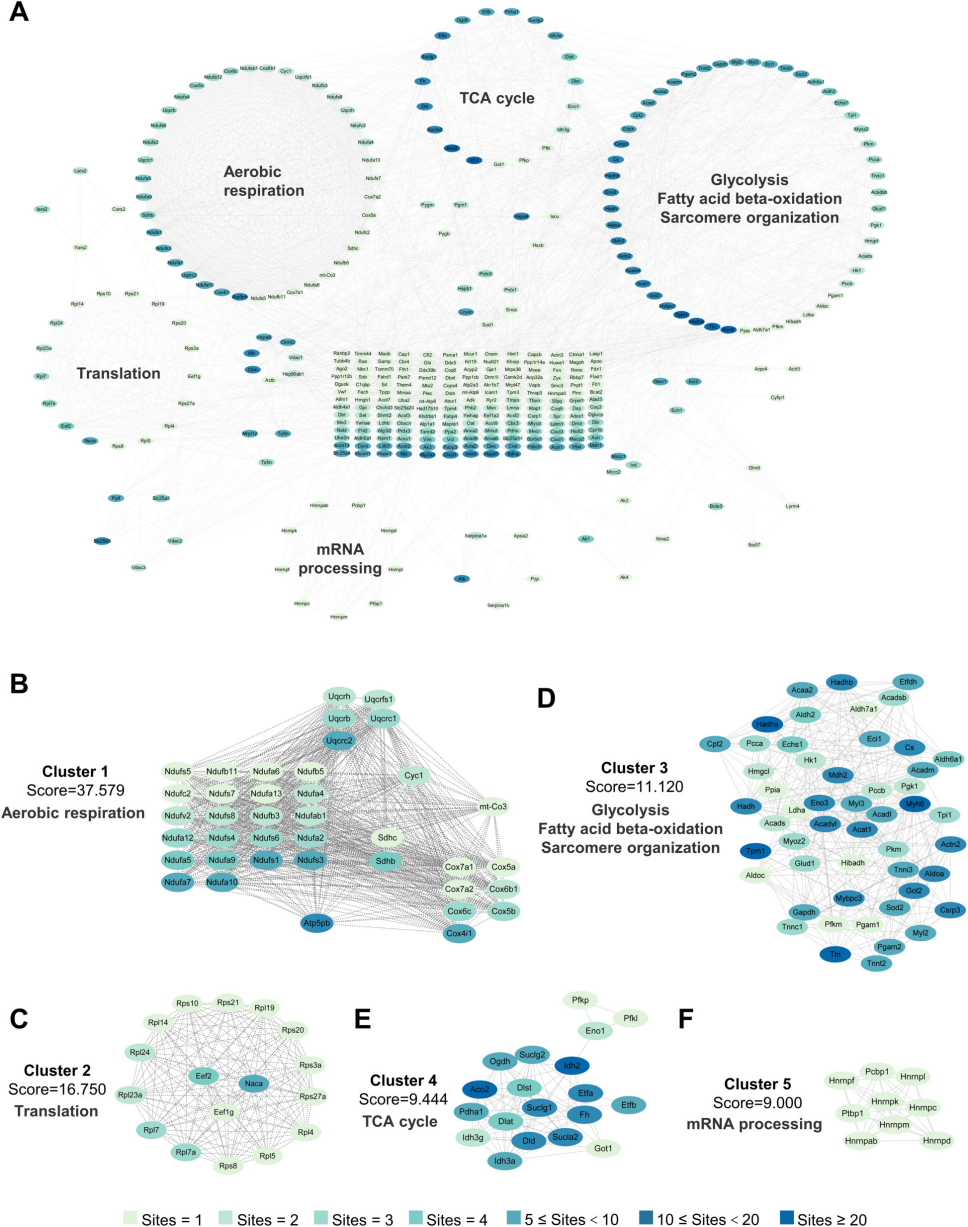
Interaction network of lactylated proteins in healthy heart. **(A)** PPI network on the basis of the STRING database (v12.0) and visualized in Cytoscape. **(B–F)** The highly connected five clusters were defined by MCODE and displayed using Cytoscape, including Aerobic respiration **(B)**, Translation **(C)**, Glycolysis, fatty acid beta-oxidation and sarcomere organization **(D)**, TCA cycle **(E)** and mRNA processing **(F)** PPI, protein-protein interaction; MCODE, molecular complex detection.

Although lactate levels increase after MI, it can also be produced in the healthy heart, and lactylation occurs under normal conditions, as confirmed in our previous experiments. The lactylation of cardiac proteins under physiological conditions and its potential regulatory functions have not been described. Therefore, we first attempted to identify all the Kla sites in the healthy heart. A total of 1,674 Kla sites were identified from 477 proteins in mouse hearts (Figure 3B, Supplementary Material S3). To investigate the Kla motifs, we compared the amino acids surrounding Kla sites against the mouse proteome and found that a overrepresentation of positively charged lysine (K) at many positions (−10, −9, −8, −7, −6, −5, −4, −3, +6, +7, +8, +9 and +10), also a notable overrepresentation of nonpolar alanine (A) at many positions (−10, −9, −8, −7, −6, −5, −4, −3, −2, −1, +1, +2, +3 and +4), while negatively charged glutamic acid (E) and nonpolar glycine (G) were largely overrepresented at the +3 position or −2, −1, 1, +1, +2, +3 and +5 positions, respectively (Figure 3C). Nearly half of the proteins (44.03%) had only one Kla site and a small proportion of proteins (1.26%) had more than 20 Kla sites (Figure 3D). Myosin-6 and titin, two important cytoskeletal proteins, have 83 and 77 Kla sites separately, which are far more than other identified proteins (Supplementary Material S4). We performed cellular compartment analysis of all lysine-lactylated proteins identified from mouse heart tissue by using a predictive tool (Wolf PSORT), and found that these proteins were widely distributed in the nucleus, mitochondria, cytoplasm, plasma membrane, and extracellular space, suggesting that lactylation modification might regulate biological functions not limited to a specific cellular sublocation, but potentially involved in various cellular compartments (Figure 3E). Thus, to understand the biological functions of lysine-lactylated substrates, we performed enrichment analysis with the GO annotation database. Our data showed that lysine-lactylated proteins were significantly enriched in cellular metabolic process with specific enrichment in proton motive force-driven mitochondrial ATP synthesis (adjusted *P* = 1.13 × 10^−39^), fatty acid metabolic process (adjusted *P* = 1.83 × 10^−24^), aerobic respiration (adjusted *P* = 7.01 × 10^−23^), and tricarboxylic acid (TCA) cycle (adjusted *P* = 6.31 × 10^−21^). Analysis of the PFAM domain database showed that Lys-lactylated proteins were enriched for acyl-CoA dehydrogenase, PDZ domain-containing protein, and biotin-requiring enzyme (Figure 3F). To understand the cellular pathways involving lysine lactylation, we performed enrichment analysis of KEGG pathways. Lysine-lactylated proteins were largely associated with metabolic pathways (adjusted *P* = 4.41 × 10^−42^), diabetic cardiomyopathy (adjusted *P* = 1.24 × 10^−41^), carbon metabolism (adjusted *P* = 4.20 × 10^−38^) and oxidative phosphorylation (adjusted *P* = 3.91 × 10^−37^) (Figure 3G). We constructed and visualized the protein-protein interaction (PPI) networks of lysine-lactylated proteins using the STRING v12.0 database (15). Our data set revealed an extensive, highly interconnected network with clusters of nodes forming distinct protein complex patterns (Figure 4A). Using the MCODE tool (16), we identified a number of highly connected subnetworks among lysine-lactylated proteins, including aerobic respiration (cluster 1, score = 37.579), translation (cluster 2, score = 16.750), glycolysis, fatty acid beta-oxidation and sarcomere organization (cluster 3, score = 11.120), TCA cycle (cluster 4, score = 9.444), and mRNA processing (cluster 5, score = 9.000) (Figures 4A–F).

### 3.4 Identification of changed Kla post-MI

**Figure 5:**
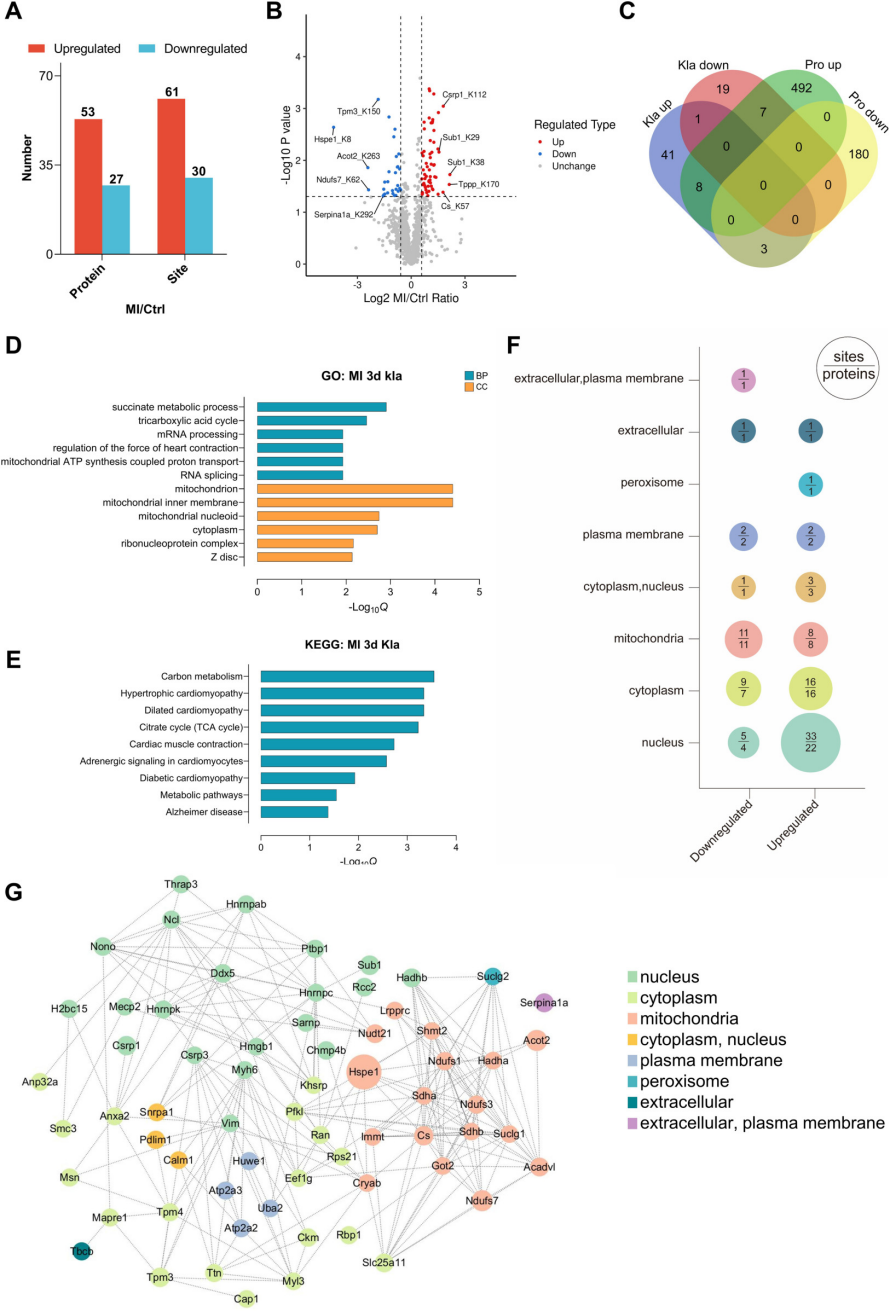
Proteomic profiling of changed Kla sites following MI. **(A)** Numbers of Kla sites and proteins exhibiting remarkable different Kla levels following MI. **(B)** Volcano plot visually displayed the number and fold-change distribution of altered Kla sites. **(C)** Venn diagram showing the relationship between proteins exhibiting remarkable Kla changes and proteins exhibiting remarkable changes in contents. **(D–E)** GO enrichment analysis **(D)** and KEGG pathway analysis **(E)** of proteins containing altered Kla sites. **(F)** Cellular compartment distribution of differentially expressed Kla sites (numbers above the line) and lactylated proteins (numbers below the line). **(G)** PPI network diagram of proteins exhibiting remarkable Kla changes on day 3 post MI.

We next compared the changes in Kla levels between control and MI groups. Compared to the control group, an average Kla levels above 1.5 or below 0.67, together with *P* value < 0.05, was considered as a significant change (Supplementary Material S5). In total, 61 sites across 53 proteins exhibited a remarkable increase in Kla levels, and 30 sites across 27 proteins exhibited a remarkable decrease in Kla levels (Figures 5A,B). The venn diagram showed that 79% of the 53 proteins exhibiting increased Kla sites and 74% of the 27 proteins exhibiting decreased Kla sites, did not have changes in protein contents (Figure 5C). The above results indicated that Kla modification might play roles post-MI independent of protein-level changes. To identify the pathophysiological processes involved in these proteins that undergo Kla-modified alteration, we performed GO and KEGG analyses. As shown in Figure 5D, proteins exhibiting Kla changes were largely associated with succinate metabolic process, TCA cycle, mRNA processing, regulation of the force of heart contraction, mitochondrial ATP synthesis coupled protein transport and RNA splicing. The cellular components in which these proteins were involved included mitochondrion, cytoplasm, ribonucleoprotein complex and Z disc (Figure 5D). Consistently, KEGG analyses also showed that proteins exhibiting Kla changes were largely associated with carbon metabolism, TCA cycle and cardiac muscle contraction (Figure 5E). Totally, lactylation may affect energy metabolism, RNA synthesis, and cytoskeleton after MI in mouse heart.

Cell compartment analysis was performed to probe the subcellular localization of proteins containing differentially lactylated lysine residues (Figure 5F). According to the prediction by Wolf PSORT, the upregulated lactylated proteins were found to be more represented in the nucleus in the ischemic hearts, hinting that the distribution of lactate and Kla modifications suggestive of a healthy or ischemic heart might have different propensity for subcellular localization. We then performed PPI analysis and observed that complicated interactions existed between most of the lactylated proteins, suggesting that Kla modifications exert a broad regulatory role by affecting a group of proteins with specific functions (Figure 5G).

### 3.5 Kla levels of various metabolic enzymes were extensively altered post-MI

**Figure 6:**
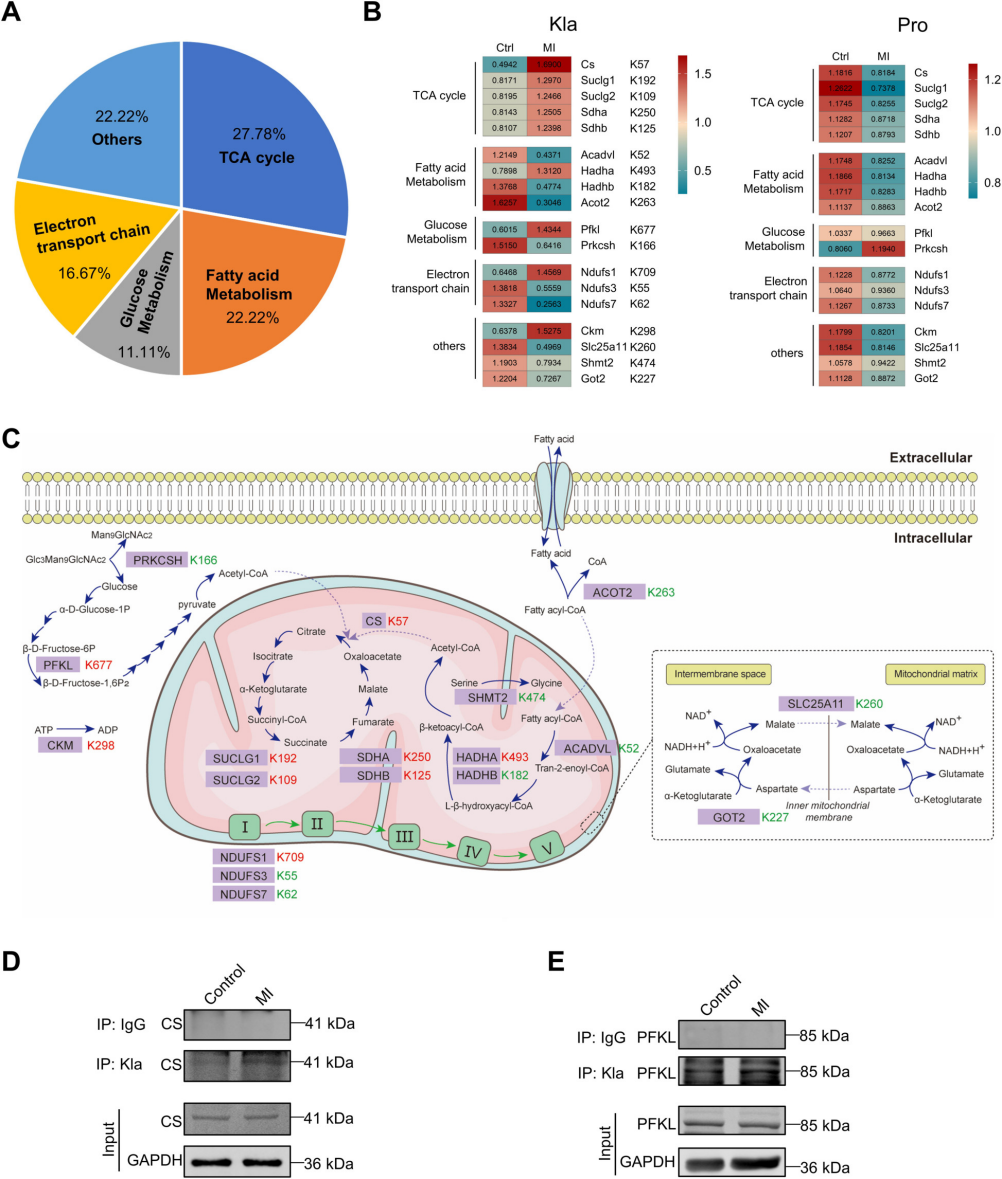
Kla changes of metabolism enzymes following MI. **(A)** Functional distribution of metabolism enzymes showing significant Kla changes post-MI. **(B)** Heatmap showing Kla changes (left) and protein-content changes (right) in metabolism enzymes on day 3 post MI. **(C)** Overview of metabolic pathways, with altered Kla sites in the infarcted heart indicated in comparison to control hearts (red represents upregulation, and green represents downregulation). **(D–E)** The Kla abundance of selected proteins (CS and PFKL) detected by immunoprecipitation and western blotting.

Many metabolic enzymes exhibited extensive Kla changes following MI and were associated mostly with TCA cycle, fatty acid metabolism, glucose metabolism and respiratory electron (Figure 6A). Two heatmaps separately displayed all the altered Kla sites of these metabolic enzymes and their protein levels between control and MI groups (Figure 6B). As mentioned earlier, Kla changes and protein-level changes might not be consistently altered. For example, CS, SUCLG1, SUCLG2, SDHA and SDHB, five important enzymes that participant in TCA cycle, showed upregulated Kla sites respectively, whereas their protein levels decreased after MI (Figure 6B). To better visualize the changed Kla sites of metabolic enzymes, and metabolic processes that were influenced, we drew a summary diagram showing overview of metabolism pathways based on proteome and lactylome (Figure 6C). Kla changed of CS and PFKL, representing proteins involved in TCA cycle and glucose metabolism, was validated using IP and WB (Figures 6D,E).

### 3.6 Kla levels of cytoskeletal proteins were extensively altered post-MI

**Figure 7:**
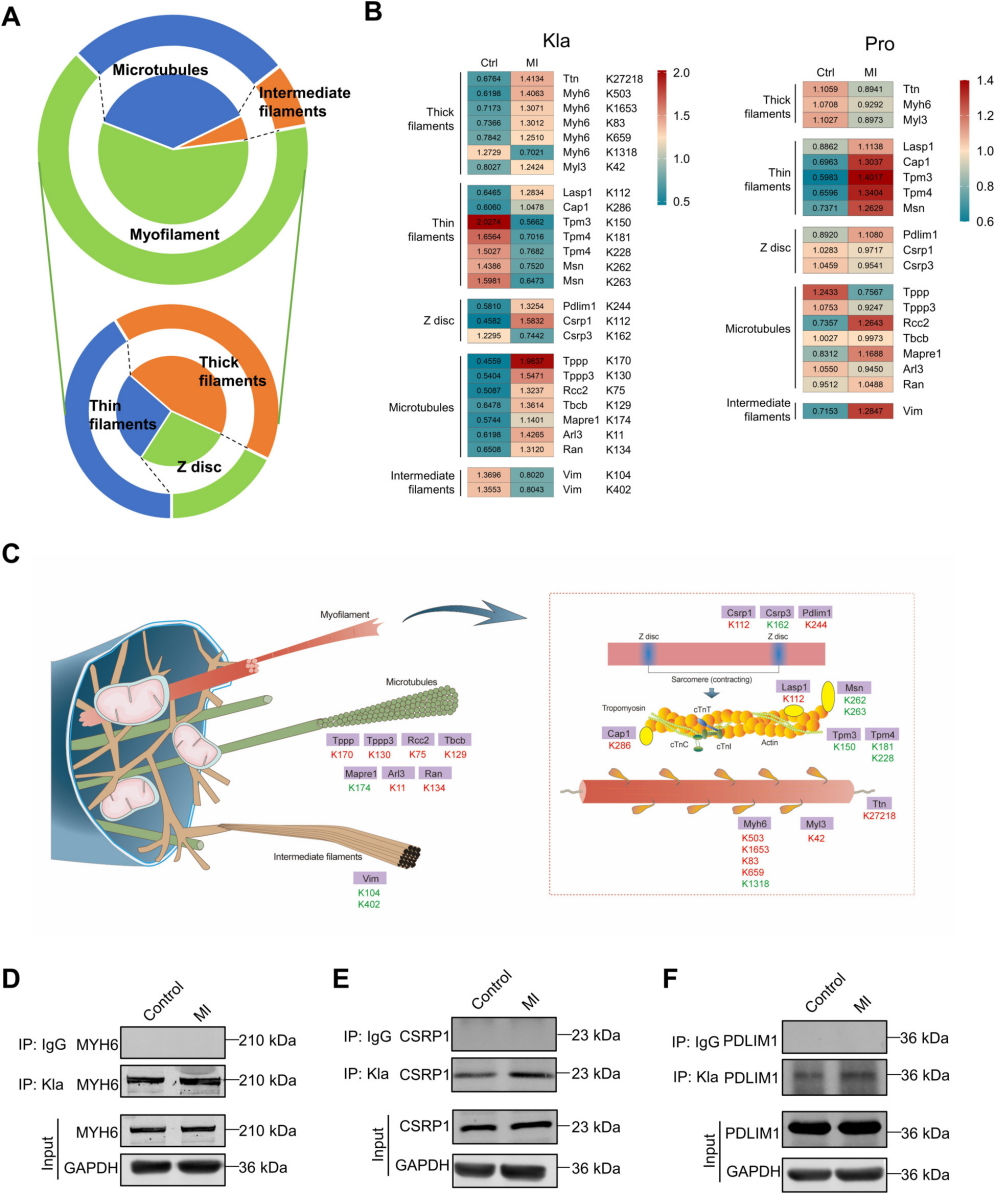
Kla changes of cytoskeletal proteins following MI. **(A)** Functional distribution of cytoskeletal proteins showing significant Kla changes post-MI. The lower part of the figure indicates subgroups of myofilament proteins. **(B)** Heatmap showing Kla changes (left) and protein-content changes (right) in cytoskeletal proteins on day 3 post MI. **(C)** Schematic diagram demonstrating significant differential Kla changes in cytoskeletal proteins post-MI (Red indicates a significant increase in Kla, while green indicates a significant decrease in Kla). **(D–F)** The Kla abundance of selected proteins (MYH6, CSRP1 and PDLIM1) detected by immunoprecipitation and western blotting.

Cytoskeletal proteins, which include myofilaments, microtubules, and intermediate filaments, are essential for maintaining cell shape and structural integrity, regulating rhythmic myocardial contractility, driving cell division and participating in cell signaling transduction (17). Following MI, cytoskeletal proteins showed extensively altered Kla, particularly myofilament proteins (Figure 7A). We also drew two heatmaps separately to display all the altered Kla sites and protein-level changes of these cytoskeletal proteins between control and MI groups (Figure 7B). Among proteins of thick filament, MYH6 exhibited most altered Kla sites, perhaps as it's the main structure protein of cardiomyocytes (Figure 7B). Moreover, seven microtubule proteins, including TPPP, TPPP3, RCC2, TBCB, MAPRE1, ARL3 and RAN exhibited a consistent increase in Kla modifications (Figure 7B). An overview of Kla-level changes of cytoskeletal proteins based on proteome and lactylome was shown in Figure 7C. Kla of a selected subset (MYH6, CSRP1 and PDLIM1) was validated using IP and WB (Figures 7D–F).

### 3.7 Kla levels of various RNA binding proteins were extensively altered post-MI

**Figure 8:**
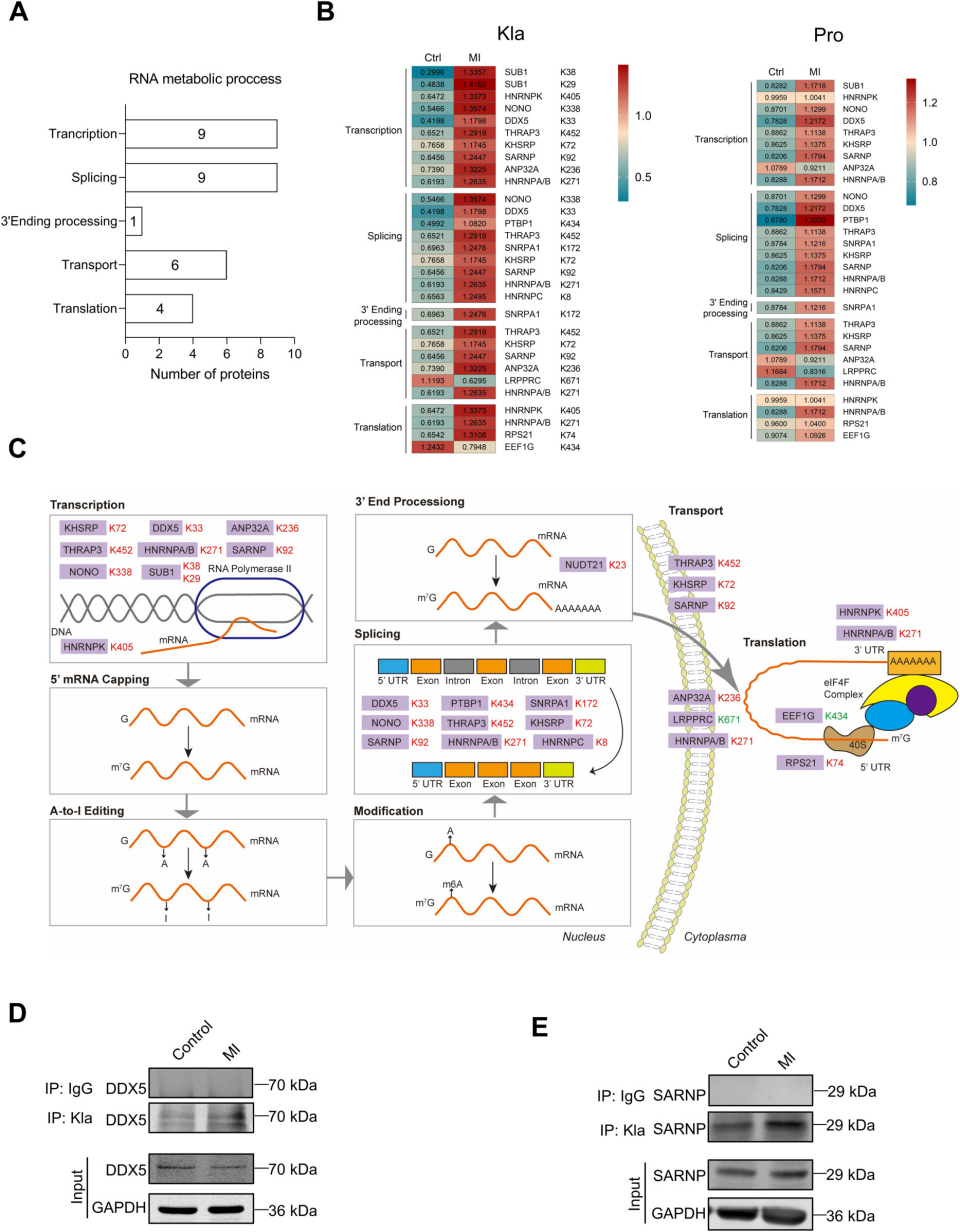
Kla changes of RNA binding proteins following MI. **(A)** Functional distribution of RNA binding proteins showing significant Kla changes post-MI. **(B)** Heatmap showing Kla changes (left) and protein-content changes (right) in RNA binding proteins on day 3 post MI. **(C)** Overview of RNA metabolic process, with altered Kla sites in the infarcted heart indicated in comparison to control hearts (Red represents upregulation, and green represents downregulation). **(D,E)** The Kla abundance of selected proteins (DDX5 and SARNP) detected by immunoprecipitation and western blotting.

Based on Kla omics analyses, we noticed that a series of proteins which participate in RNA metabolism exhibited significant changes of Kla levels. RNA metabolism contains any events in the life cycle of RNA molecules, including synthesis, processing, transport, translation and degradation (18). Our results demonstrated that proteins exhibiting extensive Kla alteration were associated with RNA translation, splicing, 3′end procession, nuclear-to-cytoplasmic transport and translation (Figure 8A). It's worth noting that all proteins that play roles in the events that happens in nucleus, including transcription, splicing and 3′end processing, showed increases in their Kla levels (Figures 8B,C). Our previous cell compartment analysis results indicated that the increased lactylated proteins were found to be more represented in the nucleus in the ischemic hearts. The above facts suggest that lactylation modifications of the infarcted heart occurred more often in the nucleus. We selected DDX5 and SARNP as representative proteins to verify their Kla changes using IP and WB (Figures 8D,E).

## 4 Discussion

Lactylation, a recently discovered PTM, is closely linked to cellular metabolism. After the onset of MI, local ischemia and hypoxia lead to an increase in glycolysis, resulting in lactate accumulation. Under the catalysis of enzymes such as P300, GCN5 and HBO1, a lactyl group can be directly attached to lysine residues on proteins, thereby altering the function of both histone and non-histone proteins (7, 9, 19). Our study directly shows that the overall level of protein lactylation increases post-MI, but this increase declines over time, indicating that the heart gradually adapts to the ischemic and hypoxic conditions. In different heart disease states, overall lactylation exhibits dynamic changes, with the lactylation of certain key proteins influencing disease progression through modulation of protein function. For example, She et al. (2024) reported an upregulation of global lactylation levels in cardiomyocytes after ischemia-reperfusion (I/R) injury, where lactylation of the mitochondrial protein MDH2 induces ferroptosis, leading to mitochondrial dysfunction (20). Zhang et al. (2023) found a downregulation of protein lactylation levels in heart failure, particularly the lactylation of α-MHC K1897, which affects the binding of α-MHC and titin, exacerbating Ang II-induced heart dysfunction in mice (12). However, to our knowledge, there has been no systematic report on the characteristics of lactylation modifications in the heart under physiological conditions, nor an analysis of global protein lactylation changes in post-MI hearts. Therefore, we designed relevant lactylome analyses and provided a comprehensive and guiding cardiac protein lactylation modification map.

First, we characterized the features of lactylation in the uninjured heart. She et al.'s omics data (2024) identified 1,026 Kla sites in 238 proteins in mouse hearts, while Zhang et al.'s data (2023) identified 576 Kla sites in 159 proteins (12, 20). In our omics data, we identified a total of 1,674 Kla sites in 477 proteins, which is a larger number than previously reported, likely due to improvements in detection technology. Among all the proteins undergoing lactylation modifications, nearly 75% of the proteins have only 1–3 Kla sites, while two proteins have more than 20 Kla sites. These two proteins are Myosin-6 and titin, dominant cytoskeletal proteins in cardiomyocytes. A possible reason for this is that these two proteins are not only abundant in cardiomyocytes but also have large molecular weights and a high number of amino acids (21). Mouse myosin-6 consists of 1,938 amino acids and has a molecular weight of 223.565 kDa. Titin, which ranks as the third most abundant protein in muscle after myosin and actin, is the largest protein known to date (22). Its length varies between approximately 27,000 and 35,000 amino acids, depending on the splice variant. Consistent with the study by She et al. (2024), our lactylation proteomic data also detected the Kla modification at the MDH2 K241 site (20). The Kla modification at the Myosin-6 K1897 site, which was reported by Zhang et al. (2023) to be crucial for the dynamic interaction between Myosin-6 and Titin, was also identified in our Kla omics. The downregulation of the Kla modification at Myosin-6 K1897 is an important mechanism that driving Ang II-induced heart failure (12). However, in our proteomic results, we found no significant differences in the levels of Kla modifications at the MDH2 K241 and Myosin-6 K1897 sites between control and MI groups, suggesting that these sites may not be key molecular mechanisms in MI progression. This indicates that the functions of lactylation sites differ across various heart diseases. To investigate the cellular localization of lactylated proteins, we performed cellular compartment analysis and found that lactylated proteins were not confined to a specific subcellular location but are widely distributed, which may be closely related to the metabolic pathways of lactate. After lactate is produced, it has multiple fates and can be exchanged between cells and the extracellular matrix, as well as across the inner and outer mitochondrial membranes, via monocarboxylate transporters (MCTs) and lactate dehydrogenase (LDH) (5). Glycolysis occurs in the cytoplasm, where pyruvate is converted to lactate by LDHA, and lactate is then exported to the extracellular matrix by MCT4 (23). Oxidative cells take up lactate through MCT1, and lactate is converted back to pyruvate in the cytoplasm by LDHB (24, 25). Pyruvate is then transported into the mitochondria by MCT1, where it enters TCA cycle to support energy metabolism (26). This lactate shuttle helps facilitate intercellular lactate sharing and links glycolysis to aerobic oxidation. Lactate itself can also directly enter the mitochondria, where it participates in regulating oxidative phosphorylation (27). Furthermore, lactate can enter the nucleus and regulate the level of histone lactylation, thus modulating gene expression (7). In our study, we observed that lactylation is constitutively present in the cytoplasm, mitochondria, nucleus, plasma membrane, and extracellular space under physiological conditions, which aligns with the widespread spatial distribution of lactate. Interestingly, lactate is a product of cellular metabolism, and proteins undergoing lactylation modifications are primarily concentrated in metabolism-related pathways, such as mitochondrial ATP synthesis, fatty acid metabolism, aerobic respiration, and TCA cycle. This may suggest that lactate is involved in a feedback regulation mechanism of cellular metabolism. In primary T cells, lactate can enter the mitochondrial matrix, stimulating the activity of the mitochondrial electron transport chain (ETC), increasing ATP synthesis, and inhibiting glycolysis, which promotes cell proliferation under glucose-limiting conditions (27). However, the authors did not address protein lactylation in their mechanistic discussion. Since our research shows widespread lactylation of proteins related to mitochondrial ATP synthesis, fatty acid metabolism, aerobic respiration, and TCA cycle, further investigation is needed to determine whether these modifications are associated with the regulation of enzyme levels and activities, whether they participate in maintaining normal cellular metabolism, and whether they play a role in responding to pathological stimuli.

Next, we investigated which Kla sites were altered after the occurrence of MI. Among the 91 altered Kla sites we identified, the majority (61 sites) showed increased Kla levels. This trend aligns with our experimental observations of an overall increase in lactylation levels. Combining proteomics data analysis, we found that these changes in Kla modifications did not appear to be broadly associated with changes in protein levels, suggesting that Kla modification more likely affected pathological processes through regulating protein function rather than content. Further enrichment analysis revealed that the proteins with altered Kla modifications could be classified into three main categories: metabolism enzymes, cytoskeletal proteins, and RNA binding proteins. Therefore, we created detailed maps of the lactylation changes in these three categories post-MI.

The altered Kla modifications in metabolism enzymes are distributed across processes like glucose metabolism, ETC, fatty acid oxidation, and TCA cycle. Interestingly, key enzymes involved in TCA cycle, including CS, SUCLG1, SUCLG2, SDHA, and SDHB, showed a significant decrease in protein levels after MI, but each of them had upregulated Kla sites, including CS K57, SUCLG1 K192, SUCLG2 K109, SDHA K250, and SDHB K125. Studies have reported that activation of TCA cycle in cardiomyocytes can improve myocardial salvage and functional outcomes following I/R injury (28). However, after cardiac injury, when macrophages sense apoptotic cells or damaged tissue and drive phagocytosis, the TREM2-SYK-SMAD4 signaling pathway is activated, inhibiting the transcription of SLC25A53. Low expression of SLC25A53 leads to reduced NAD transport efficiency, creating a bottleneck in TCA cycle and releasing anti-inflammatory organic acids (such as oxaloacetate). Interestingly, this disruption of the TCA cycle actually leads to higher myocardial repair efficiency after MI (29). Therefore, the role of various metabolic enzymes in MI is cell-specific, and this should be considered when studying the impact of lactylation on metabolic enzymes in MI.

Among the myocardial cytoskeletal proteins, the most significant changes in Kla modification were observed in myofilament proteins. Myosin-6 in the thick filaments had five altered Kla sites, including K503, K1653, K83, K659, and K1318. The protein level of Myosin-6 decreased in the heart after MI, which is consistent with our proteomics results (30). Interestingly, promoting Myosin-6 synthesis has beneficial effects on MI. Studies have shown that inhibiting Myosin-6 depletion induced by oxygen-glucose deprivation improves inflammation and apoptosis in HL-1 mouse cardiomyocytes (31). Transgenic rabbits with sustained expression of Myosin-6 showed better cardiac function after MI (32). However, research on the PTMs of Myosin-6 and their relevance to MI or other cardiovascular diseases is still limited. The decrease in Kla modification at Myosin-6 K1897 site impairs the binding of Myosin-6 and Titin, exacerbating heart failure damage (12). Since Myosin-6 is one of the key cytoskeletal proteins in cardiomyocytes, extending our proteomics study to further investigate the role of Myosin-6 lactylation in MI would have significant scientific potential.

In RNA metabolism, RNA binding proteins involved in transcription, splicing, 3′ ending process, transport, and translation showed extensive changes in Kla modification, especially in nuclear processes like transcription and splicing. DDX5 can activate the β-catenin signaling pathway, leading to the transcriptional activation of cyclin D1 and c-Myc, promoting cardiomyocyte regeneration after MI (33). Our study identified Kla modification at the K33 site of DDX5, and this modification increased after MI. Based on our proteomics results, we can select important RNA binding proteins in the heart and study the functional effects of lactylation at specific sites and their impact on MI. From another perspective, we can also examine the impact of global changes in lactylation modifications of RNA binding proteins on cardiomyocytes. Although overall lactylation levels and protein levels do not show a consistent trend, we found that the lactylation levels of all identified RNA splicing regulatory proteins, including NONO, SNRPA1, SARNP, and members of the hnRNP family, were increased, along with their protein levels. Alternative splicing plays an important role in MI. For example, alternative splicing of IGF-1 pre-mRNA results in several isoforms with distinct E-domains. Among them, the minor isoform IGF-1Eb, also referred to as Mechano-Growth Factor (MGF), is predominantly expressed in the heart following MI in mice. Both systemic and heart-specific delivery of the MGF E-domain peptide have been shown to improve contractile function and prevent pathological remodeling of the heart after MI (34). Therefore, future research could explore the connection between lactylation, alternative splicing and MI to identify new therapeutic targets for MI.

Our study has several limitations: (1) In investigating the alterations in protein lactylation in a mouse model of MI, we utilized only male mice. Future studies should include both sexes to explore potential sex-specific effects. (2) The analysis of the subcellular localization changes of lactylation modifications under physiological conditions or following MI was solely based on a prediction tool. Further experimental validation is warranted. (3) All proteins with altered lactylation levels identified in this study require further experimental validation to confirm the modification changes, their impact on protein function, and their roles in MI.

## 5 Conclusion

This study establishes the first comprehensive lactylome atlas encompassing both physiological and post-MI cardiac states, revealing that lactylation are widely present in the heart, primarily affecting metabolic pathways. Additionally, following MI, due to ischemia and hypoxia, glycolysis is activated, leading to a slight increase in lactylation levels, particularly impacting metabolism enzymes, cytoskeletal proteins, and RNA binding proteins. Our study provides a guiding perspective on lactylation in the heart, and this comprehensive characterization of Kla may offer novel mechanistic insights for MI progression and potential therapeutic targeting.

## Data Availability

The datasets presented in this study can be found in online repositories. The names of the repository/repositories and accession number(s) can be found in the article/Supplementary Material.
