# Supplementary material for: Characterization of protein lactylation in healthy and ischemic mouse hearts
Source: Front Cardiovasc Med. 2025 Sep 29;12:1644886. doi: 10.3389/fcvm.2025.1644886 (PMC12515869; doi:10.3389/fcvm.2025.1644886)
Supplement: Supplementary file 6 [file Datasheet1.pdf]

Supplementary Material : Raw WB

Figure 2A

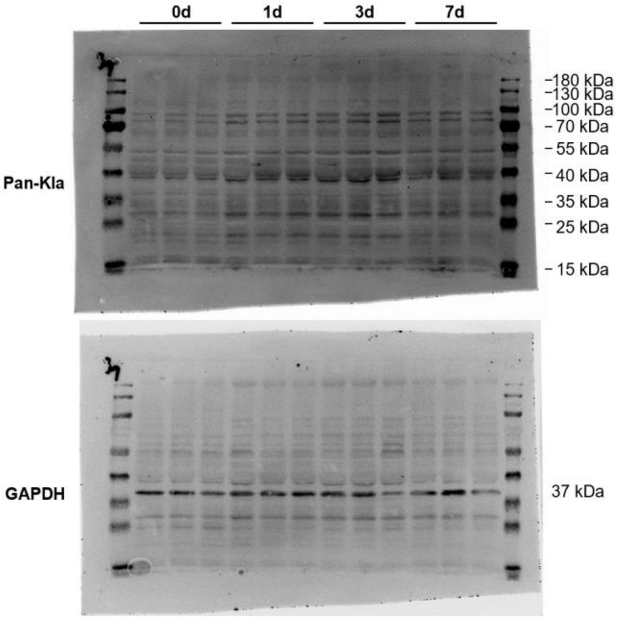

Figure 6D

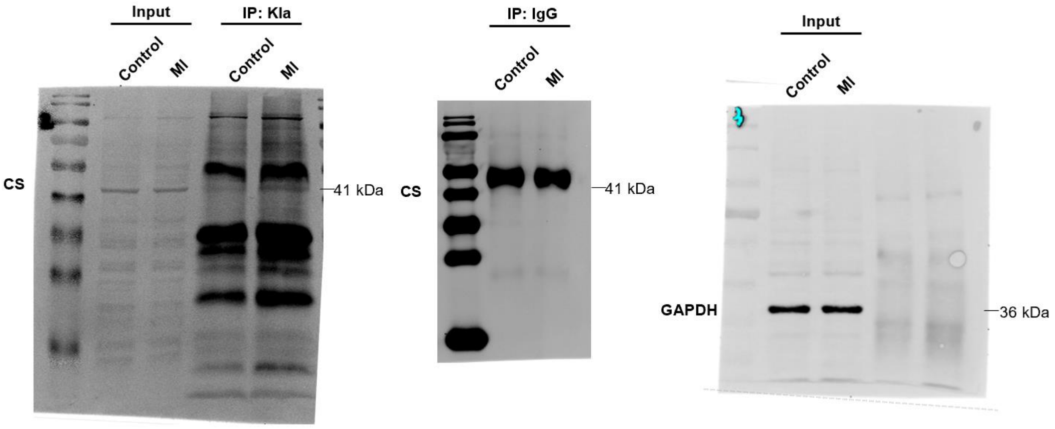

Figure 6E

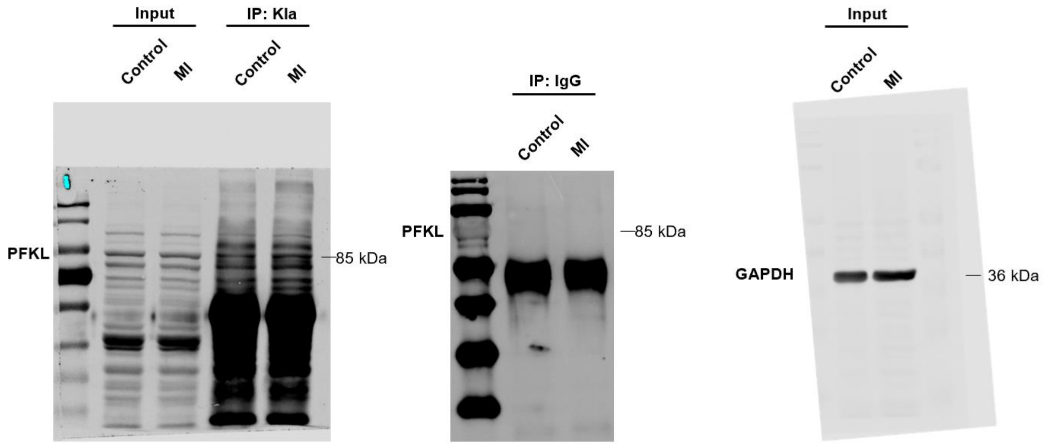

Figure 7D

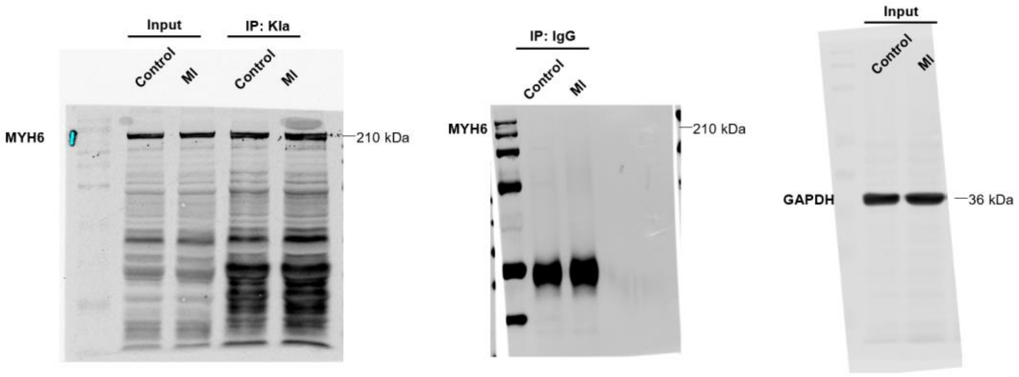

Figure 7E

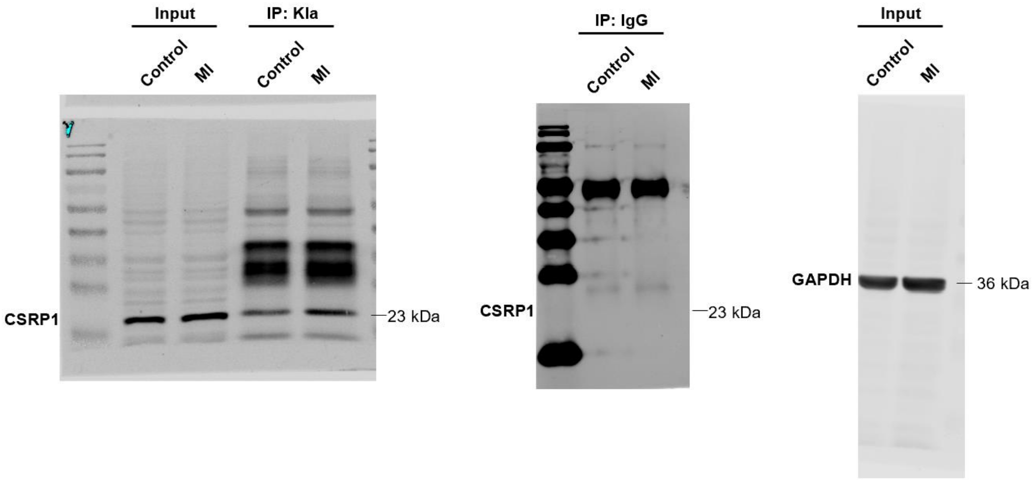

Figure 7F

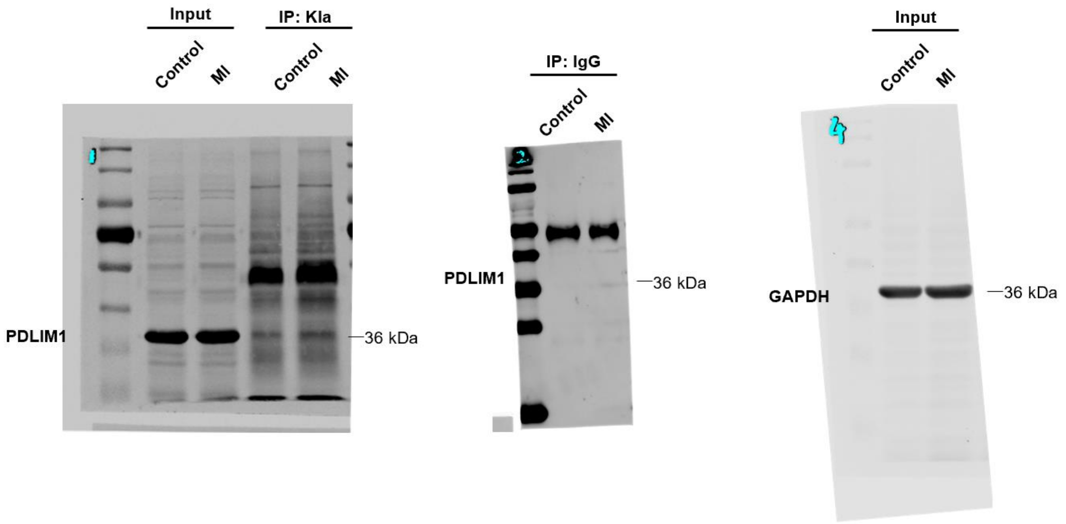

Figure 8D

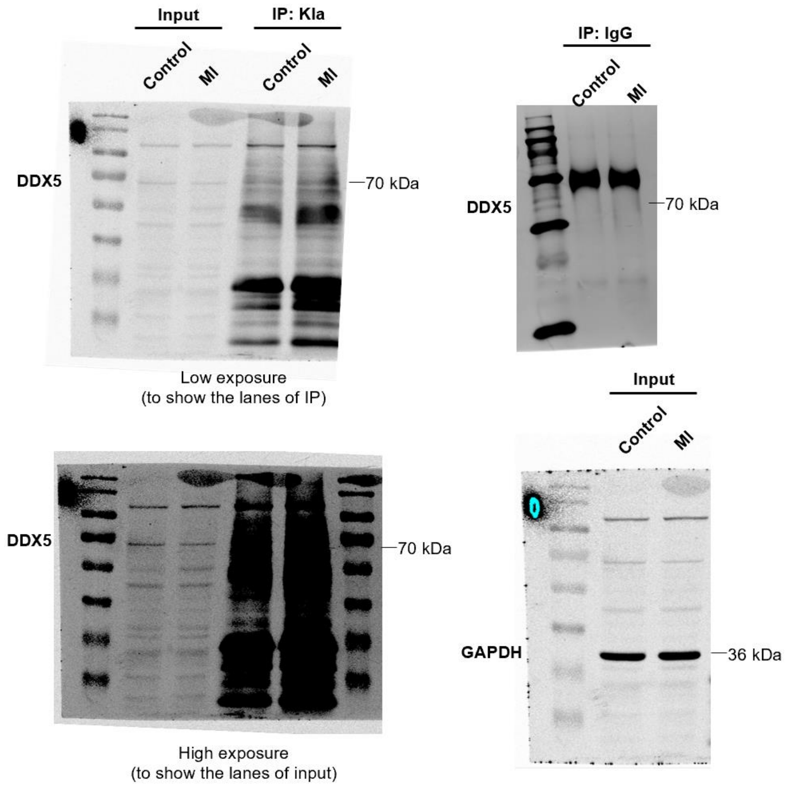

Figure 8E

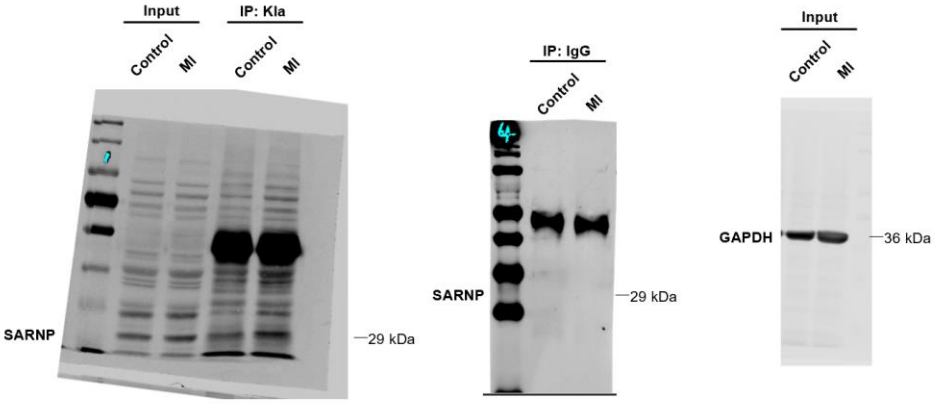
Input
IP: Kla
Control
MI
Control
MI
SARNP
29 kDa
IP: IgG
Control
MI
SARNP
29 kDa
Input
Control
MI
GAPDH
36 kDa
