# Supplementary figures and images for: Characterization of protein lactylation in healthy and ischemic mouse hearts
Source: Front Cardiovasc Med. 2025 Sep 29;12:1644886. doi: 10.3389/fcvm.2025.1644886 (PMC12515869; doi:10.3389/fcvm.2025.1644886)

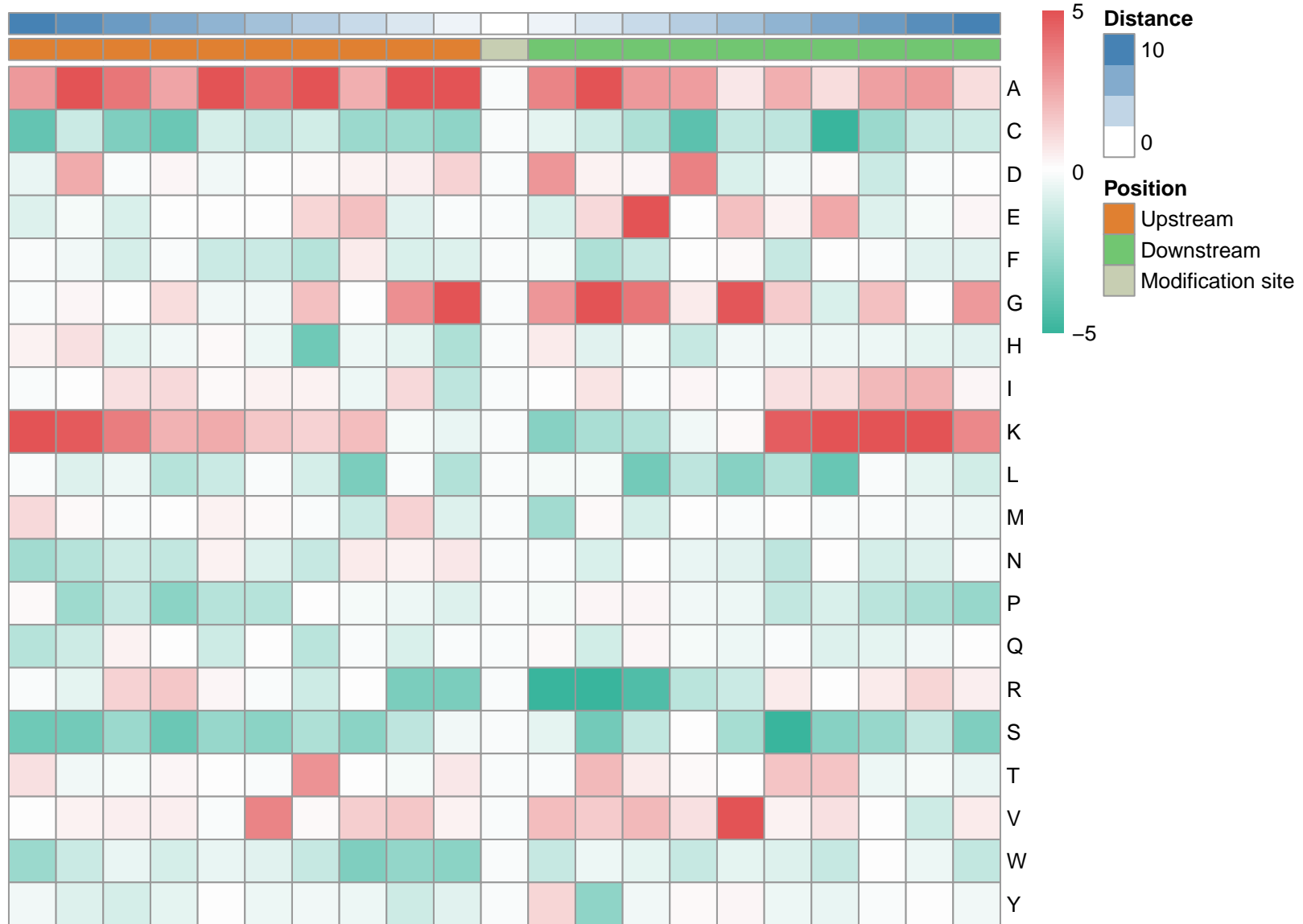

Supplement: Supplementary file 7 [file Image2.pdf]

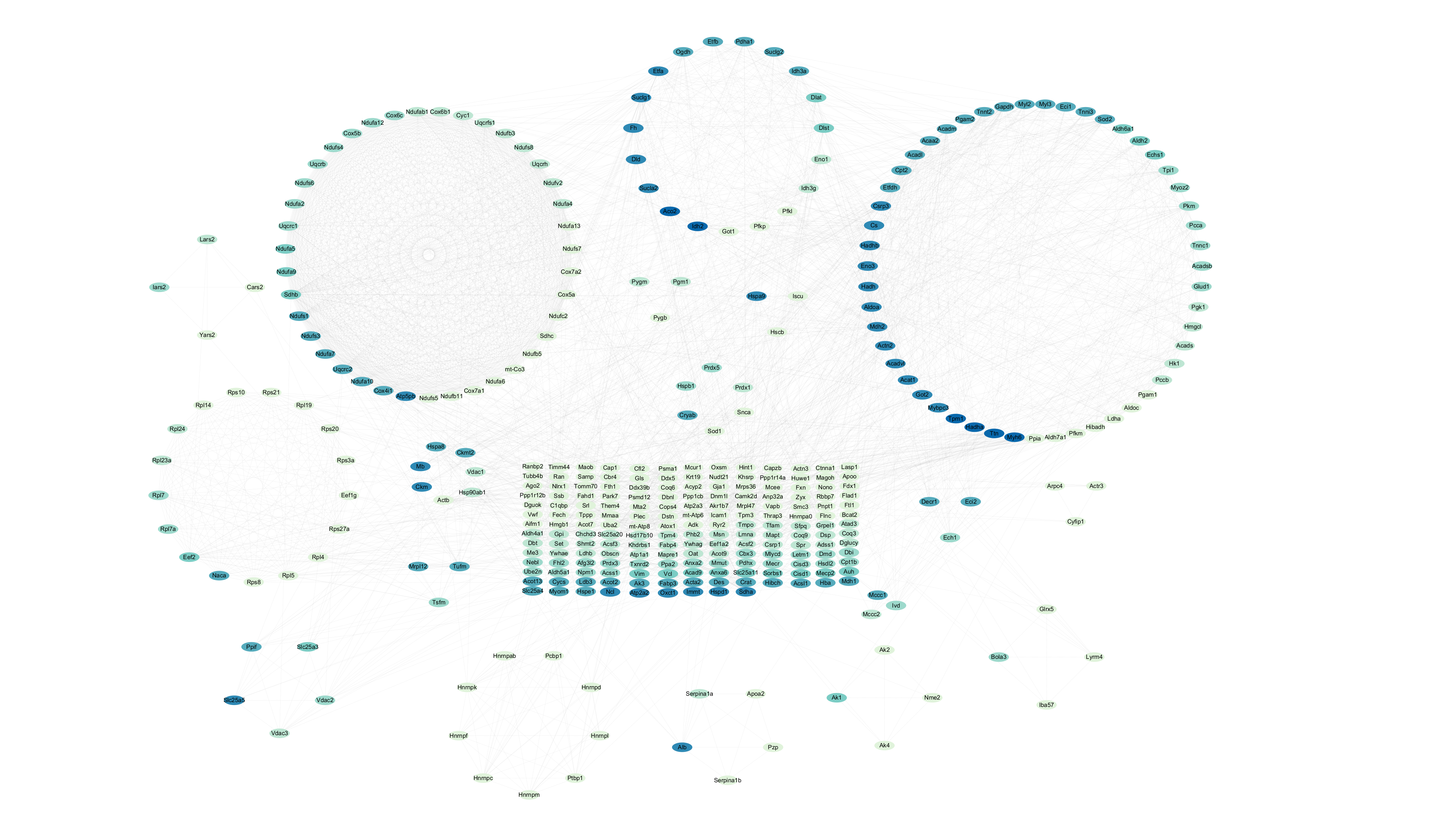

Supplement: Supplementary file 8 [file Image1.png]
